# Selecting targets for the diagnosis of *Schistosoma mansoni* infection: An integrative approach using multi-omic and immunoinformatics data

**DOI:** 10.1371/journal.pone.0182299

**Published:** 2017-08-17

**Authors:** Gardenia B. F. Carvalho, Daniela M. Resende, Liliane M. V. Siqueira, Marcelo D. Lopes, Débora O. Lopes, Paulo Marcos Z. Coelho, Andréa Teixeira-Carvalho, Jeronimo C. Ruiz, Cristina T. Fonseca

**Affiliations:** 1 Biologia e Imunologia de Doenças Infecciosas e Parasitária, Instituto René Rachou, Fiocruz Minas, Belo Horizonte, Minas Gerais, Brazil; 2 Rede Fiocruz Minas de Identificação e Produção de Antígenos–RIPAG, Belo Horizonte, Minas Gerais, Brazil; 3 Programa de Pós-graduação em Biologia Computacional e Sistemas, Instituto Oswaldo Cruz, Fiocruz, Rio de Janeiro, Rio de Janeiro, Brazil; 4 Informática de Biosistemas, Instituto René Rachou, Fiocruz Minas, Belo Horizonte, Minas Gerais, Brazil; 5 Biologia do *Schistosoma mansoni* e sua interação com o hospedeiro, Instituto René Rachou, Fiocruz Minas, Belo Horizonte, Minas Gerais, Brazil; 6 Laboratório de Biologia Molecular, Universidade Federal de São João Del-Rei, Divinópolis, Minas Gerais, Brazil; 7 Grupo Integrado de Pesquisas em Biomarcadores, Instituto René Rachou, Fiocruz Minas, Belo Horizonte, Minas Gerais, Brazil; McGill University, CANADA

## Abstract

In order to effectively control and monitor schistosomiasis, new diagnostic methods are essential. Taking advantage of computational approaches provided by immunoinformatics and considering the availability of *Schistosoma mansoni* predicted proteome information, candidate antigens of schistosomiasis were selected and used in immunodiagnosis tests based on Enzime-linked Immunosorbent Assay (ELISA). The computational selection strategy was based on signal peptide prediction; low similarity to human proteins; B- and T-cell epitope prediction; location and expression in different parasite life stages within definitive host. Results of the above-mentioned analysis were parsed to extract meaningful biological information and loaded into a relational database developed to integrate them. In the end, seven proteins were selected and one B-cell linear epitope from each one of them was selected using B-cell epitope score and the presence of intrinsically disordered regions (IDRs). These predicted epitopes generated synthetic peptides that were used in ELISA assays to validate the rational strategy of *in silico* selection. ELISA was performed using sera from residents of areas of low endemicity for *S*. *mansoni* infection and also from healthy donors (HD), not living in an endemic area for schistosomiasis. Discrimination of negative (NEG) and positive (INF) individuals from endemic areas was performed using parasitological and molecular methods. All infected individuals were treated with praziquantel, and serum samples were obtained from them 30 and 180 days post-treatment (30DPT and 180DPT). Results revealed higher IgG levels in INF group than in HD and NEG groups when peptides 1, 3, 4, 5 and 7 were used. Moreover, using peptide 5, ELISA achieved the best performance, since it could discriminate between individuals living in an endemic area that were actively infected from those that were not (NEG, 30DPT, 180DPT groups). Our experimental results also indicate that the computational prediction approach developed is feasible for identifying promising candidates for the diagnosis of schistosomiasis and other diseases.

## Introduction

Schistosomiasis remains one of the most prevalent parasitic diseases in the world with more than 240 million people infected in 78 countries [1]. Control strategies have been based on chemotherapy, but these attempts have failed to interrupt transmission. Part of this failure could be attributed to the absence of an accurate method of diagnosis that is able to determine the real prevalence of the disease in populations, and that could monitor the success of interventions and assess healing after therapeutic intervention [2–4]. Parasitological tests are still the most widely used diagnostic methods of schistosomiasis control programs [5,6], and of these, the Kato-Katz technique is the most used due to its low cost, abillity to detect different helminths infection and greater sensibility in áreas high intensity infections [7–9]. However, low parasite burdens require examination of more slides or association of the parasitological tests with serological and molecular techniques to have an accurate diagnosis of the disease. In fact, molecular and immunological techniques have proven to be more sensitive and promising in identifying infection in individuals with negative coproscopic results [10–15].

An example of an immunological technique that has provided satisfactory results in the diagnosis of schistosomiasis is the urine-based point-of-care (POC-CCA) assay for detecting circulating cathodic antigen in urine samples (Rapid Medical Diagnostics, Pretoria, South Africa). This test has demonstrated promise for use in epidemiological studies, in clinical laboratories and in endemic areas, with higher sensitivity than the Kato-Katz technique [16–19]. However, more studies are necessary to assess the efficacy of this technique in the field, especially in areas of low endemicity [20,21].

Immunodiagnostic methods based on serology have been widely used and have greater sensitivity than parasitological methods [6,22–24], particularly in areas of low endemicity [25,26]. Among serological tests, ELISA assay is widely used for the diagnosis of schistosomiasis, but one of the difficulties in using this method is the choice of the parasite`s antigen. Crude antigens may exhibit cross-reactivity with other helminthes, as well as possess low sensitivity [27]. To overcome this obstacle, purified and recombinant antigens [28–35] have been used.

Immunoinformatics emerged as a new multidisciplinary approach for identifying diagnostic targets at the beginning of 21^st^ century, with the accumulation of genomic data in public domain databases. Indeed, some bioinformatic tools have already been used to identify *Schistosoma* antigens. *In silico* analyses were used to search for protein tandem repeats in the genome of *S*. *mansoni*, and seven diagnostic candidates were identified as a result of this study [36]. Guo et al. (2012) used a bioinformatics analysis to find target sequences for molecular diagnosis from *S*. *japonicum* retrotransposons [37]. Also working with *S*. *japonicum*, Zhang et al. (2007) searched for B-cell epitopes in three pre-selected proteins in order to design two multi-epitope chimeric proteins to be used as diagnostic targets, and showed that these proteins were able to react with sera of *S*. *japonicum* infected patients [38]. Immunoinformatics has also been used to identify diagnostic targets of other eukaryotic pathogens, such as the use of proteomics combined with B-cell epitope search to find potential antigens from *Cryptococcus gattii* proteome [39].

In this context, in a previous work, our research group has scanned the predicted proteome of *S*. *mansoni* using tools from SchistoDB 2.0 [40] and online bioinformatic`s tools. From these previous studies we were able to identify six potential diagnostic candidates [41], among which was Sm200, a 200 kDa tegumental protein. Although this protein was highly sensitive in identifying infected patients from endemic areas, it was not able to differentiate between non-infected and infected individuals from an area of low endemicity [42].

Besides the classical immunoinformatics tools, such as epitope prediction and subcellular location prediction, another approach that has emerged for identifying immunogenic proteins is the search for B-cell epitopes associated with structurally disordered regions. In fact, it has already been demonstrated that immunogenic peptides may be closely associated with flexibility [43], which could promote the exposure of these immunogenic regions to the immune system. This strategy also takes into account that intrinsically disordered regions (IDRs) were present in approximately 60–70% of the proteins from the eukaryotics’ predicted proteomes analyzed, and are responsible for important biological functions [44].

Based on the above, the present work was developed due to the necessity to find a target to be used in a test for the diagnosis of schistosomiasis with higher specificity and sensitivity than the tests currently used, and also able to identify cured individuals after chemotherapy. The developed and implemented strategy involves epitope prediction, associated with subcellular location and expression in different stages of *S*. *mansoni*, to find proteins from the whole predicted proteome of the parasite that could be used as diagnostic targets. In addition, a relational database was created to integrate all of the data generated. From the proteins selected, peptides were submitted to manual curation and selected based on their score in B-cell epitopes prediction and on the presence of IDRs in the neighborhood of the epitope location in the protein. These peptides were synthesized and evaluated experimentally by ELISA in order to validate the computational prediction.

## Methods

### Genomic data

13,273 transcripts from *S*. *mansoni* were obtained from SchistoDB 2.0 [40]. Data from protein expression linked with different parasite life cycle stages in human host, including egg, schistosomula, lung schistosomula, and adult worm, were also downloaded for computational downstream analysis.

### Epitope prediction

All proteins from the parasite *S*. *mansoni* were screened for B-cell and TCD4+ epitopes. For B-cell epitope prediction, we used BepiPred 1.0 [45], AAP12 [46] and BCPred12 [47]; and for TCD4+ epitope prediction, we used NetMHCII 2.2 [48], which predicts the affinity of epitopes to 17 different alleles.

### Prediction of intrinsic disordered proteins (IDPs)

Prediction of protein disorder was based on the consensus prediction obtained by the following algorithms: DisEMBL [49], GlobPlot [50], IUPred [51], and VSL2B [52]. A region of a protein was considered disordered when 40 or more consecutive aminoacids were predicted as disordered, since prediction accuracy was shown to be higher in longer disordered regions [53].

### Prediction of subcellular location

It was also important to investigate the subcellular location of the proteins, since this information is of great significance to the functional analysis of the proteins themselves and also of specific regions. For this, the following algorithms were selected: Sigcleave [54], TargetP 1.1 [55]and SignalP 4.0 [56] to verify the presence of signal peptides; TMHMM 2.0 [57,58] to verify transmembrane domains; and SherLoc2 [59] to verify subcellular location.

### Sequence similarity searches

The Standalone BLAST (Basic Local Alignment Search Tool) algorithm [60] was used to perform sequence similarity searches of pre-selected diagnostic targets from *S*. *mansoni* against predicted human proteins. This analysis is necessary in order to avoid cross-reaction with proteins from host organisms.

### Workflow of analysis for selection of target diagnostic proteins

**Fig 1:**
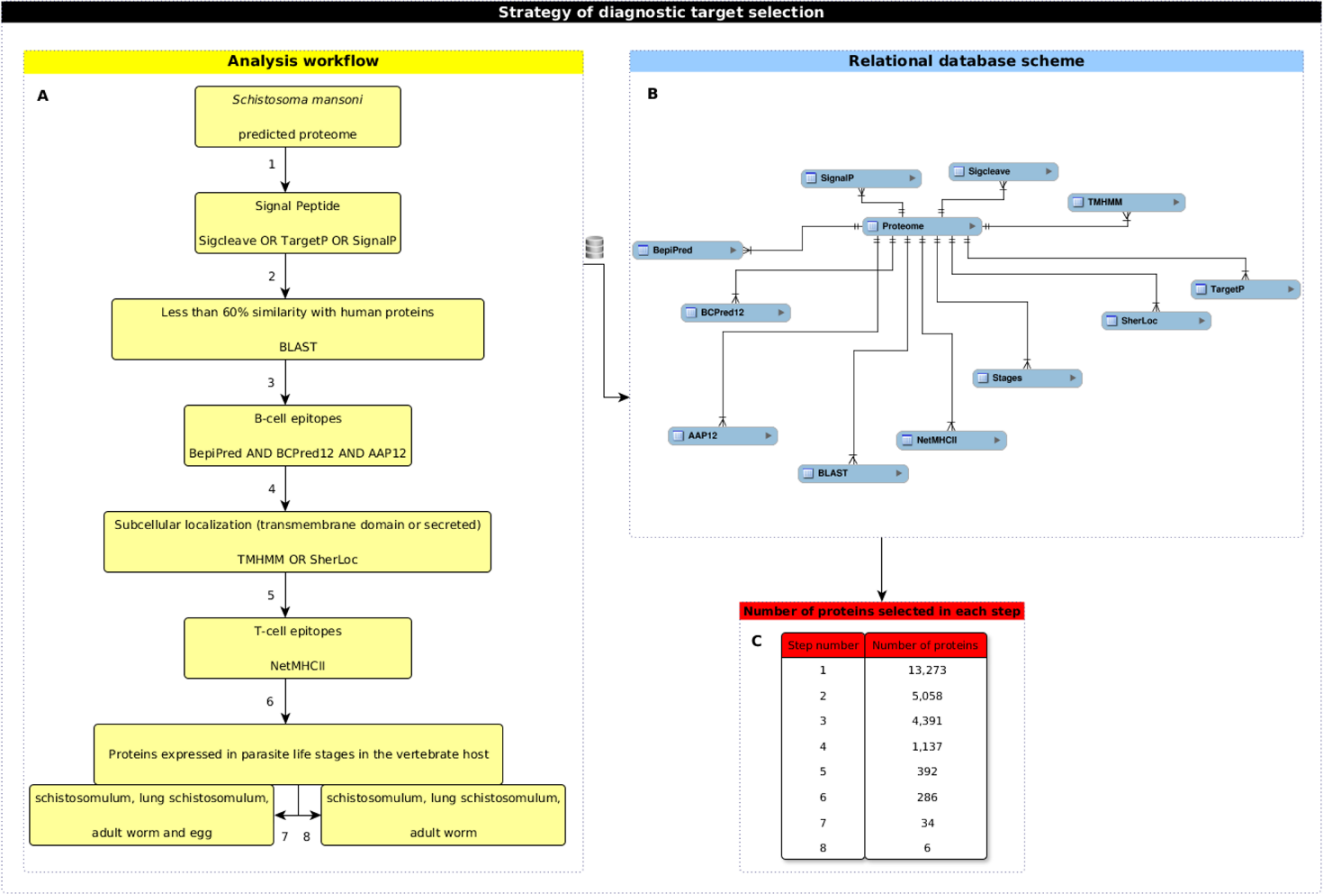
Strategy of diagnostic target selection. (A) Each step of the workflow analysis was numbered according to the strategy used for target selection. (B) Schematic representation of the relational database developed to integrate data obtained after computational predictions. (C) Number of proteins selected after each step from (A).

All predictions described above were performed on local servers and loaded into the computational analytical workflow developed in order to select potential diagnostic antigens (Fig 1A). The first step of our approach involved selection of proteins with signal peptides as predicted by Sigcleave, TargetP 1.1 or SignalP. Next, we used the BLAST algorithm to exclude proteins with more than 60% of similarity with human predicted proteome. After these analyses, we selected proteins with at least one linear B-cell epitope predicted by BepiPred 1.0, BCPreds, and AAP12. Epitopes with prediction scores above one on these three predictors were chosen. Proteins harboring the predicted epitopes were subsequently submitted to subcellular location prediction with SherLoc2 and scanned for transmembrane domains with TMHMM 2.0. Target proteins predicted at the extracellular compartment or at plasmatic membrane were selected. These target proteins were analyzed for the presence of TCD4+ epitopes with affinity to 17, 16, 15, 14 or 13MHCII alleles predicted by NetMHCII 2.2. Finally, in order to use the information regarding the parasite life cycle stages on the definitive host, two different strategies were applied. In the first one, proteins predicted as simultaneously expressed on schistosomulum, lung schistosomulum, adult worm and egg were selected. In the second one, proteins simultaneously expressed in all the above stages except egg were chosen.

### Data integration

Data from *S*. *mansoni* predicted proteome, from parasite life cycle stages, and from all predictions performed were automatically integrated in a MySQL relational database (Fig 1B). This database was developed in order to make it possible to extract biologically relevant information related to diagnostic targets using the previously described workflow analysis.

### Selection of peptides

Custom peptide synthesis was performed using the sequence information from the predicted epitopes from the proteins selected by the workflow described above.

The strategy of peptides’ selection was based on the following steps: a) sequences predicted as linear B-cell epitopes by AAP12 and BCPreds with a score of at least 1, and by BepiPred 1.0 with a score of at least 1.5; b) if the analyses failed to find a peptide, a second query was performed considering just AAP12 and BepiPred 1.0, and the same scores described above; and c) if no epitope has been found yet, the cutoff scores were changed to at least 0.9 for AAP12 and at least 1 for BepiPred 1.0. After the selection was finished, an additional filtering step was performed removing epitopes with the following characteristics: a) located at predicted signal peptides regions; b) located at predicted transmembrane domains regions; and c) predicted as identical to human epitopes.

In the final step, we preferentially selected epitopes containing IDRs in their neighborhood and predicted by BCPreds, even with a lower score. After selection of the epitopes, we performed another BLAST step against host`s genome and all the proteins from *Ancylostoma ceylanicum*; *A*. *duodenale*; *Ascaris lumbricoides*; *Enterobius vermicularis*; *Hymenolepis nana*; *Necator americanus*; *Taenia solium*; *T*. *saginata*; *Trichuris trichiura* available in NCBI database to avoid similar peptides.

### Synthesis of peptides

Peptides were identified with numbers from 1 to 7 in order to facilitate the description of results. Peptide 1 was synthesized using PS3TM synthesizer (Protein Technologies, Inc) by chemical solid phase synthesis using the Fmoc strategy. Cleavage of peptides was carried out manually as described by Chan & White (2000)[61], and purification was performed by high performance liquid chromatography, with using a Shimadzu Prominence chromatograph. The other peptides were synthesized by the company Genescript with more than 90% purity. All peptides were diluted in DMSO (dimethyl sulfoxide) at a concentration of 1 μg/μL and stored at -70°C.

### Human serum samples

In this study, sera from 58 individuals (female/male: 24/34) from an area of low endemicity for *S*. *mansoni* infection (Pedra Preta, Minas Gerais, Brazil) and 13 sera from healthy donors (HD group) (female/male: 06/07) not living in an endemic area for schistosomiasis were used. Pedra Preta is a little village in a schistosome endemic area next do Montes Claros, MG. The sera from de individuals evaluated in our study was collected in a previous study performed by our group in a rural zone of Pedra Preta with approximately 230 residents. The previous study evaluated 201 individuals. The prevalence determined by examination of two Kato-Katz slides, which is recommended by the Brazilian Ministry of Heath to determine infection, was 8% with a mean parasite load of 40.6 opg. However a 35.8% of positivity were found when feces samples were analyzed using Kato-Katz (18 slides) and TF-Test® techniques [62]. Individuals co-infected with *Ascaris lumbricoides*, *Thricuris trichiura*, hookworm, *Taenia sp*, *Hymenolepis nana* and *Enterobius vermiculares* were observed in this endemic area, but in our study only sera of *S*. *mansoni* monoinfected individuals were used before and after treatment.

The discrimination of negative (NEG group) and positive (INF group) serum samples of individuals from endemic areas was performed according to parasitological and molecular methodology described by Siqueira et al (2015)[62]. Briefly, each participant provided four separate stool samples on each of four consecutive days for use in the Kato-Katz [7], TF-Test® [63,64] and PCR-ELISA [65] techniques. Individual serum samples were obtained after centrifugation of blood samples at 3000g for 5 minutes. These samples were kept at -20°C until moment of use. Sera samples from twenty six individuals infected with *S*. *mansoni* and from 25 individuals from endemic area with negative stool examination were evaluated.

All participants with positive stool examination for schistosomiasis determined by parasitological methods were treated with praziquantel in a single dose of 60mg/Kg for children and 50mg/Kg for adults, as recommended by the Brazilian Ministry of Health. Sera were obtained 30 and 180 days post-treatment (groups 30DPT and 180DPT), at these time points feces were also collected. Cure rate was determined by examination of 18 Kato-Katz slides and TF-Test®. Seventeen and nine sera samples were evaluated 30 and 180 days post-treatment, respectively. Some sera samples were from individuals previously evaluated in the infected group.

### Ethics

The use of both healthy donor sera and sera from individuals living in the Pedra Preta endemic area for schistosomiasis were approved by the Ethical Research Committee of the René Rachou Research Institute -Fiocruz (CEPSH/CPqRR 03/2008: 105/2004-OF.215-TEC) and the National Ethical Board (784/2008, CONEP14886). All participants and parents/legal guardians were informed about the purpose and objectives of the study before providing written informed consent to participate of this study.

### ELISA against human sera

The ELISA assay was first standardized using 1 or 2μg/ml of synthetic peptide incubated with pool of sera from individuals belonging to the healthy donor group; negative group or infected group in serial dilution in PBST beginning at 1:20 and ending at 1:1,280. The conjugate was evaluated in a dilution of 1:60,000 (anti-human IgG-HRP). After standardization, the assay was performed in flat bottom plates (Maxisorp NUNC®) sensitized with 100μl/well of carbonate bicarbonate buffer 0.05M, pH 9.6, containing peptide at a concentration of 1μg/mL (peptides 1,5,6 and 7) or 2μg/mL (peptides 2 and 3), and incubated for approximately 16 hours at 4°C. To remove the unbound proteins, the plates were washed three times with 150mM PBS containing 0.05% Tween 20 (PBST) and blocked with PBST plus 10% fetal bovine serum for 2 hours at 37°C. The sera from patients were diluted 1:40 (peptides 4 and 7), 1:80 (peptides 1 and 2), 1:160 (peptides 3 and 6) and 1:320 (peptide 5) in PBST. These sera were added (100μl/well) to the plates and incubated for 2 hours at 37°C. After washing the plates (3X), an anti-human IgG secondary antibody conjugated to peroxidase (Sigma Aldrich) was used at a dilution of 1: 60,000 in PBST for one hour at 37°C. After three washes, the reaction was revealed for 10 minutes with 100μl of liquid substrate 3, 3', 5, 5'-tetramethylbenzidine (TMB/Sigma). The reaction was stopped with 50μL of sulfuric acid and the optical density determined by an automatic ELISA reader (Multiskan, Thermo Scientific), using a filter of 450nm.

### Statistical analysis

The software package GraphPad Prism 4.0 (GraphPad Software, San Diego, CA, USA) was used for statistical analysis. Kruskal-Wallis test followed by Dunn post-test were performed on samples without a normal distribution. Receptor Operating Characteristic curves (ROC curves) were used to calculate sensitivity, specificity and the cutoff points between infected and healthy donor groups or between infected and uninfeced individuals from endemic area. Positive predictive values (PPV) and Negative Predictive Values (NPV) was determined by the following formula: PPV = number of true positives/(number of true positives + number of false positives); NPV = number of true negatives/(number of true negatives + number of false negatives)

## Results

### Selected proteins

In this study, target proteins to be used in schistosomiasis diagnosis were selected using the computational workflow described in methods section (Fig 1C). The analysis started with the predicted proteome of *S*. *mansoni*, comprised of 13,273 proteins. Only 5,058 proteins had a signal peptide predicted in its sequence and remained in the workflow. 4,391 of the proteins that remained in the workflow presented less than 60% of similarity with human proteins. Prediction of B cell epitope was the third filtering step, which resulted in a reduction of the number of proteins to 1,137. From these, 392 proteins, predicted to be located on the plasmatic membrane or to be secreted, were selected. For T CD4+ cell epitope prediction, 17 MHC alleles were analyzed (14 HLA-DR; H2-IAb, H2-IAd e H2-IAs). The adopted criterion took into account the predicted epitopes located along the complete extension of the protein with affinity to bind the maximum number of alleles. As result, none of the 392 proteins possessed epitopes with affinity to the 17 MHC alleles, five were found to have epitopes with affinity to 16 MHC alleles, 72 presented epitopes predicted to bind 15 MHC alleles, 186 presented predicted epitopes with affinity to 14 MHC alleles and 286 proteins presented epitopes with affinity to the 13 MHC alleles. Finally, in each set of proteins, we analyzed predicted profile of expression in different parasite life cycle stages within the vertebrate host. Two different approaches were used. First, proteins predicted to be expressed simultaneously on schistosomulum, lung schistosomulum, adult worm and egg stages were selected. The combination of this criterion with the criteria described above resulted in the selection of two proteins with epitopes predicted to bind 16 MHC alleles, eight proteins containing epitopes predicted to bind 15 MHC alleles, fourteen proteins presenting epitopes with affinity to 14 MHC alleles and ten proteins with epitopes predicted to bind 13 MHC alleles (S1 Table). Second, proteins predicted to be expressed simultaneously on schistosomulum, lung schistosomulum and adult worm were selected, resulting in the selection of five proteins with epitopes predicted to bind 14 MHC alleles and one protein presenting epitopes with affinity to 13 MHC alleles (S1 Table). The seven proteins with predicted epitope with affinity to bind the highest number of MHC alleles in each approach were selected. In the group of proteins predicted to be expressed simultaneously on schistosomulum, lung schistosomulum, adult worm and egg, two proteins that had predicted epitopes with affinity to 16 MHC alleles were selected. In the group of proteins predicted to be simultaneously expressed in all of the above-mentioned stages except egg, five proteins with epitopes predicted to bind 14 MHC alleles were selected (Table 1).

**Table 1:** *Schistosoma mansoni* proteins selected after *in silico* analysis, according to the strategy outlined in the workflow.

ID	Function Predicted	Amino acids Length	Life stages ^a^	Number of alleles
Smp_136560	Expressed protein	1995	1	16
Smp_141860	Heat containing protein, putative	4619	1	16
Smp_093840	Trispanning orphan receptor; TORE, putative	239	2	14
Smp_126160	Poly (p) /ATP NAD kinase, putative	1077	2	14
Smp_150390.1	Expressed protein	668	2	14
Smp_167240	Expressed protein	776	2	14
Smp_180240	F-spondin, putative	941	2	14

^a^ Parasite life stages in definitive host

1—Schistosomulum, lung chistosomulum, adult worm and egg

2—Schistosomulum, lung schistosomulum and adult worm

### Serological tests

Seven peptides (one for each selected protein), with no similarity host or other heelminths’ proteins, were chosen to be used as antigens in the serological tests. Peptide number, protein ID (systematic name), life stages in which they are expressed, peptide sequence, degree of purity, prediction of IDRs and disorder predictors for each selected peptide are described in Table 2. Three of the peptides having predicted IDRs in their neighborhood were selected.

**Table 2:** Epitopes selected to be used as synthetic peptides in ELISA test.

Peptide Number	ID (Initial and final coordinates)	Life stages^a^	Sequence of peptide	Degree of purity (%)	Prediction of IDRs (Initial and final coordinates)	Disorder predictors
1	Smp_136560 (1564–1578)	1	ITEGNNSREGNSEKV	60.1	1501–1658	REM465, GlobPipe e IUPred
2	Smp_141860 (1694–1709)	1	NHSMDKDDDDFSDIDK	95,31	1693–1793	REM465, GlobPipe, IUPred e VSL2B
3	Smp_093840(219–233)	2	TTTNKDDTQINSAPS	96,69	182–239	REM465, GlobPipe, IUPred e VSL2B
4	Smp_126160(438–452)	2	LVTPESKYYSSLPGN	95,97	-	-
5	Smp_150390.1(216–230)	2	SLPSNAHNNDNNSSD	95,55	-	-
6	Smp_167240(213–228)	2	QCDLDTQWNPAGTEYS	97,74	-	-
7	Smp_180240(339–353)	2	RDWPTTLTGAGGSTT	97,67	-	-

^a^ Life stages of parasite in definitive host

1 –Schistosomulum, lung chistosomulum, adult worm and egg

2—Schistosomulum, lung schistosomulum and adult worm.

**Fig 2:**
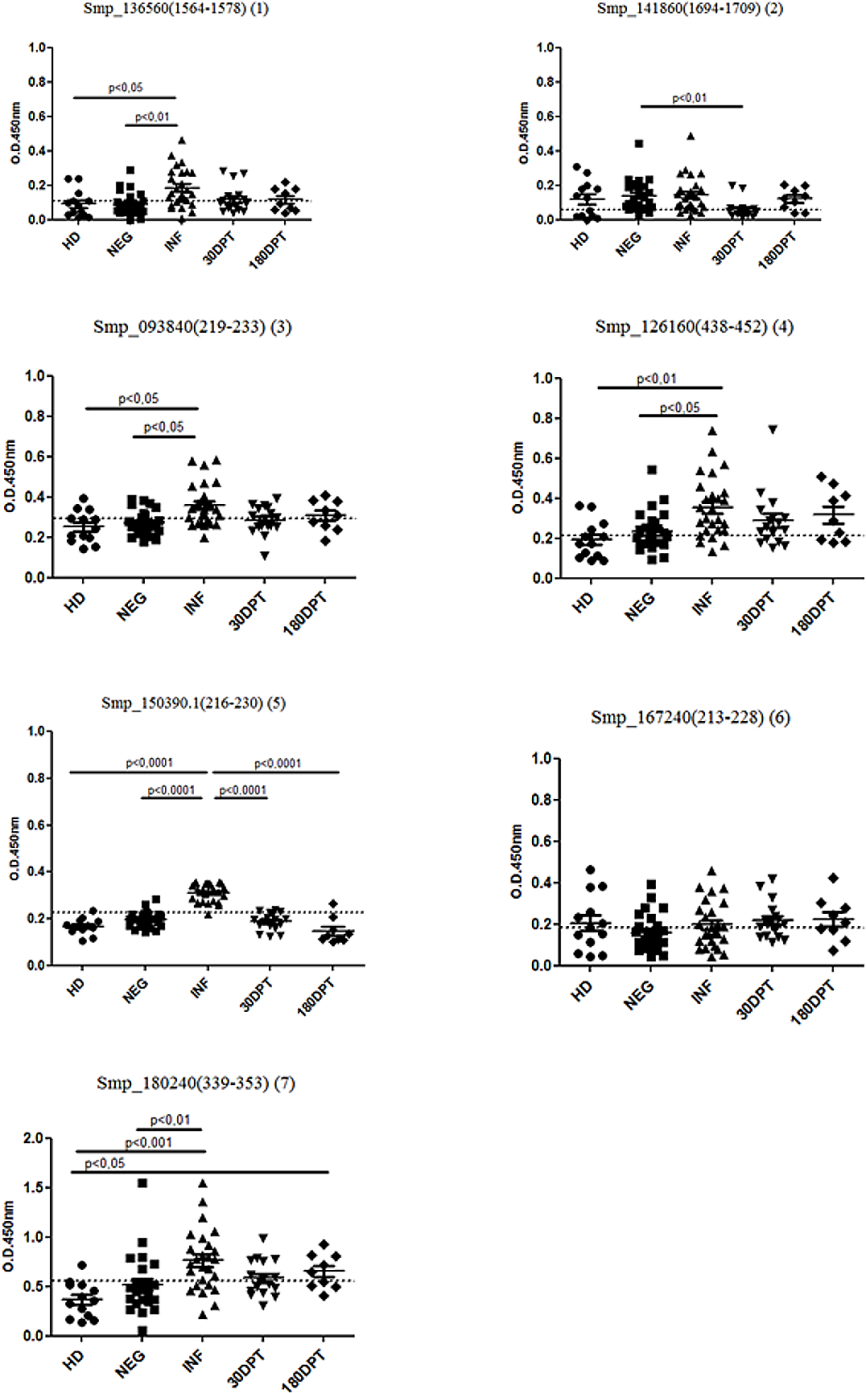
Human IgG-specific response against synthetic peptides from *S*. *mansoni* targeted for use in diagnosis. Serum samples were collected from 51 individuals living in an area of low endemicity for *S*. *mansoni* infection. Samples from 25 individuals with negative stool examination (NEG) and from 26 individuals with positive stool examination were evaluated (INF). Sera from patients treated 30 and 180 days after treatment were also evaluated. Additionally, sera from 13 healthy donors (HD) not living in an endemic area for schistosomiasis were used as a negative control. Significant differences between groups are indicated in the graphic. Dashed lines represent the cutoff in the absorbance level which determines specificity and sensitivity.

These synthetically produced peptides were employed in ELISA tests against human sera from the following experimental groups: HD, NEG, INF, 30DPT and 180DPT, as described in the Materials and Methods section. Significant IgG reativity against peptides 1, 3, 4, 5 and 7 were observed in the group of *S*. *mansoni* infected individuals in comparison with healthy donor. Significant IgG levels against peptides 1, 3, 4, and 5 were also observed in infected group when compared to individuals from the endemic area with negative stool examination (Fig 2). A significant reduction in serum reactivity to peptide 5 was observed in the groups 30DPT and 180DPT (Fig 2). Reactivity to peptide 7 was still observed in sera from the 180 days post-treatment group. Paired analysis before and after treatment were performed for some individuals. Data demonstrate that a significant descrease in IgG levels against peptides 1, 3 and 5 is observed 30 and 180 days post-treatment. Decreased IgG levels against peptides 2 and 4 were also observed 30 and 180 days post-treatment, respectivelly (S1 Fig).

Specificities for the ELISA assay were 76.92%, 46.15%, 76.92%, 69.23%, 100%, 53,85% and 92,31% while sensitivities were 73.08%, 84.62%, 69.23%, 84.62%, 96.15%, 53.85% and 73.08% for peptides 1, 2, 3, 4, 5, 6 and 7, respectively when the cutoff point was determined using the data from healthy donor and infected groups (S2 Table). Similar results were observed when cutoff point was determined using the data from uninfected individuals of endemic area (S3 Table) The performance of each peptide used as an antigen in the ELISA can be observed in the ROC curve (S2 and S3 Figs) and in S2 and S3 Tables, which summarizes important performance values.

## Discussion

Currently, schistosomiasis transmission in Brazil is maintained by individuals with low levels of infection, and so there is a need to develop effective approaches for prevention, control and elimination of this disease [66]. A diagnostic test with high specificity and sensitivity would be an important tool for limiting the transmission of this parasite, potentially resulting in its control and elimination. Diagnostic tests with low sensitivity may not detect individuals with low-levels of infection, who then remain infected and contribute substantially to schistosomiasis transmission.

The strategy that we used to select potential proteins for immunodiagnostic assays was based on criteria linked to important characteristics of recognition by the host immune system. These criteria included the presence of signal peptides, low similarity with human proteins, liner B and T CD4+ epitopes and favorable subcellular location (plasma membrane or secreted). We also analyzed the predicted parasite life-cycle stages in the definitive host, considering two approaches: a) selection of proteins for schistosoma diagnostic, covering all the stages in definitive host (schistosomulum, lung schistosomulum, adult worm and egg); and b) selection of proteins for post-treatment diagnosis of cure, excluding the proteins expressed in the egg stage, since after treatment the adult worm is eliminated, but the egg remains for a longer period of time, and its antigens could stimulate antibody production. Because the rational applyed for selecting antigens to be used in post-treatment diagnosis of cure, is based on the use of antigens expressed in schistsosmula and adult worm, reactivity in case of reinfection might occur. Thus making it difficult for the test to descriminate between treatment failure and reinfection. Paired analysis from data of reativicty to peptides before and after treatment suggest that cases of reinfection might have occurred in some individuals (S1 Fig).

After performing the workflow, we observed that some of the selected proteins were annotated as expressed proteins without predicted molecular function (hypothetical proteins) and consequently have been poorly studied. These proteins may be good targets for schistosomiasis serological diagnosis, since they matched all criteria of selection. Other proteins were annotated as putative and with a functional annotation that suggests their function, sometimes describing a conserved domain. These findings suggest the low quality of genome annotation linked with the parasite proteins.

Recently, recombinant antigens [41,67–69] and synthetic peptides [70] have been used in order to increase the specificity of serological tests. One recombinant antigen that has been widely used for identifying patients with low parasite burdens is CCA (circulating cathodic antigen) [69]. In the present work, we verified that this protein matched all the criteria established in our workflow, suggesting our methodology reflects a promising rationale for diagnostic. However, this protein was not selected in our study, because we decided to synthetize peptides from proteins containing epitopes predicted to bind to at least 14 different MHC alleles, and CCA contains epitopes predicted to bind only 13 different MHC alleles.

To validate our strategy of targets selection for use in schistosmiasis diagnosis, linear B cell epitopes were identified in the target proteins and synthetically produced to be tested in ELISA. The approach for epitope selection was based on the benchmark of prediction algorithms described by Resende et al. (2012), which showed that the best combination of algorithms for linear B cell epitope prediction in parasites is BepiPred and AAP12 [71]. Besides, we selected epitopes harboring IDRs in their neighborhood, since the importance of this relationship had already been shown in an immunogenic B cell epitope from *Plasmodium vivax* [43]. However, we could not confirm this for *S*. *mansoni* in our analysis since the peptide with the best performance in ELISA tests was not associated with IDRs.

For the ELISA assays, sera from individuals of an area of low endemicity for *S*. *mansoni* infection and sera from healthy donors (HD group) not living in an endemic area for schistosomiasis were used. The synthetic peptides showed a diversity of sensitivities and specificities. Among the seven peptides tested, three (peptides 1, 5 and 7) performed better, with sensitivities and specificities higher than 73%. It is important to highlight that peptide 5 had the best results, with a specificity of 100% and a sensitivity of 96.15%. Furthermore, this peptide was able to differentiate sera from individuals of an area of low endemicity before and after treatment.

Although some peptides tested here did not exhibit high sensitivity and specificity, all of them performed better than the Kato-Katz technique (S4 Table). In this study, Kato-Katz exhibited a sensitivity of 36.46% when two slides from two fecal samples were analyzed, and a sensitivity of 46.15% when 18 slides from four fecal samples were evaluated.

Other studies have also focused on searching for new antigens to schistosomiasis serological diagnosis using different approaches. Xu and collaborators (2014), using computational tools, selected and expressed a recombinant protein known as SjSP, which was tested in ELISA against human sera and had a sensitivity of 90.4% and a specificity of 98.9% [72] in the diagnosis of *S*. *japonicum* infection. Zhong and collaborators (2010) also reported good performance in ELISA testing using the *S*. *japonicum* recombinant proteins SjLAP and SjFBPA, with sensitivity ranging from 87.8% to 98.1% and 84.7% to 100%, respectively [35].

Taking into account studies based on *S*. *mansoni* recombinant proteins or peptides, our group has recently reported a recombinant protein, Sm200, with a sensitivity of 90% and a specificity of 93% in ELISA tests [42]. Another recombinant protein, SmRP26, was also used in ELISA tests for the diagnosis of *S*. *mansoni* infection in acute and chronic phases of the disease, with sensitivities of 83% and 32%, respectively, and a specificity of 97% [73]. Grenfell and collaborators used a recombinant circulating cathodic antigen (CCA) protein and two peptides derived from CCA as *S*. *mansoni* antigens in ELISA tests for the diagnosis of the disease. The results demonstrated 96% sensitivity for recombinant CCA protein and 80% and 74% for peptides 1 and 2, respectively [69]. Moreover, a pool of synthetic peptides was also tested as antigens for the diagnosis of *S*. *mansoni* infection. This ELISA test showed a sensitivity of 86.8% and a specificity of 94.2% [33]. Similar sensitivities were observed in our study using the peptides identified, but in contrast to the studies described above, we also analyzed peptide recognition by sera from individuals living in an endemic area of schistosomiasis, but characterized as non-infected by parasitological and molecular tests. In this regard, ELISA using peptide 5 achieved the best performance, since it could discriminate individuals living in endemic area who were actively infected from those who were not. Therefore an ELISA test using this peptide has the potential to be used in epidemiological surveys, as well as to monitor treatment efficiency.

In summary, we demonstrated that a bioinformatics approach was successful in selecting good candidates for use in the diagnosis of schistosomiasis, with peptide 5 being the most promising.

## Supporting information

S1 Fig Reactivity to each selected peptides in sera from infected individuals before and 30 or 180 days post-treatment. Significant diferences are pointed in the graphs.
(TIF)

S2 Fig Graphical representation of ROC Curves, calculated with data from infected and healthy donors groups, demonstrating the specificity and sensitivity of each selected peptide. (TIF)

S3 Fig Graphical representation of ROC Curves, calculated with data from infected and non-infected individuals from endemic area, demonstrating the specificity and sensitivity of each selected peptide. (TIF)

S1 Table *Schistosoma mansoni* proteins selected after *in silico* analysis according to the strategy outlined in the workflow. ^a^Life stages of parasite in definitive host.
1—schistosomulum, lung chistosomulum, adult worm and egg.
2—schistosomulum, lung schistosomulum and adult worm.
(DOCX)

S2 Table Performance of each peptide used as an antigen in ELISA using sera from healthy donors as negative control. ^a^CI—confidence interval; ^b^PPV = positive predictive value; PPV = number of true positives/(number of true positives + number of false positives); ^c^NPV—negative predictive value; NPV = number of true negatives/(number of true negatives + number of false negatives).
(DOCX)

S3 Table Performance of each peptide used as an antigen in ELISA using sera from non-infected individuals living in endemic area as negative control. ^a^CI—confidence interval; ^b^PPV = positive predictive value; PPV = number of true positives/(number of true positives + number of false positives); ^c^NPV—negative predictive value; NPV = number of true negatives/(number of true negatives + number of false negatives).
(DOCX)

S4 Table Description of parasitological, molecular and serological results from each serum sample analyzed in the study. Nd—not determined.
O.D.—optical density at 560nm.
*—serum from patient evalueted before treatament in the infected group (INF).
nd—not determined.
r—antigen-reactive sera.
(DOCX)
