# Supplementary figures and images for: Selecting targets for the diagnosis of Schistosoma mansoni infection: An integrative approach using multi-omic and immunoinformatics data
Source: PLoS One. 2017 Aug 17;12(8):e0182299. doi: 10.1371/journal.pone.0182299 (PMC5560627; doi:10.1371/journal.pone.0182299)

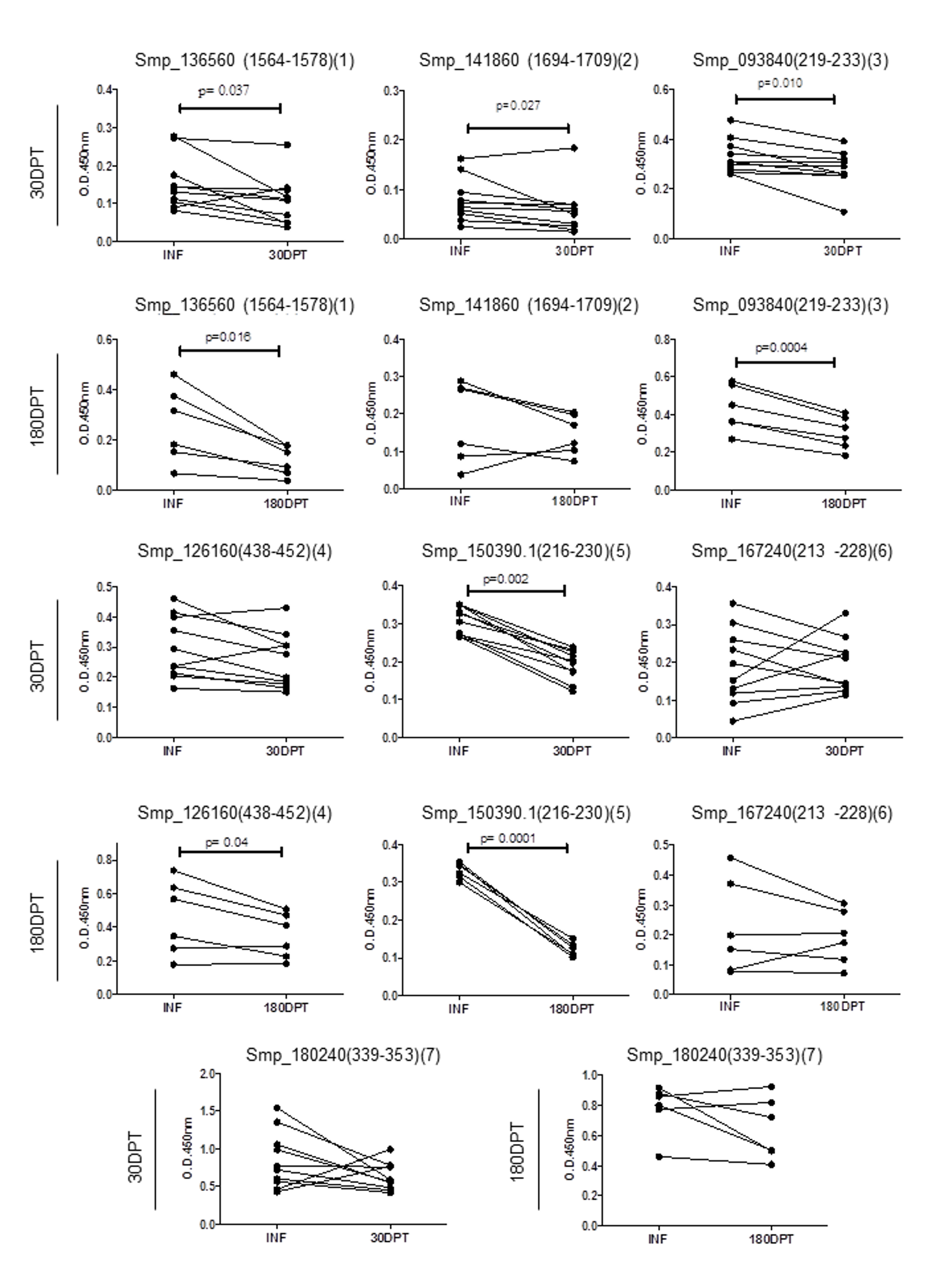

Supplement: S1 Fig — Significant diferences are pointed in the graphs. (TIF) [file pone.0182299.s001.tif]

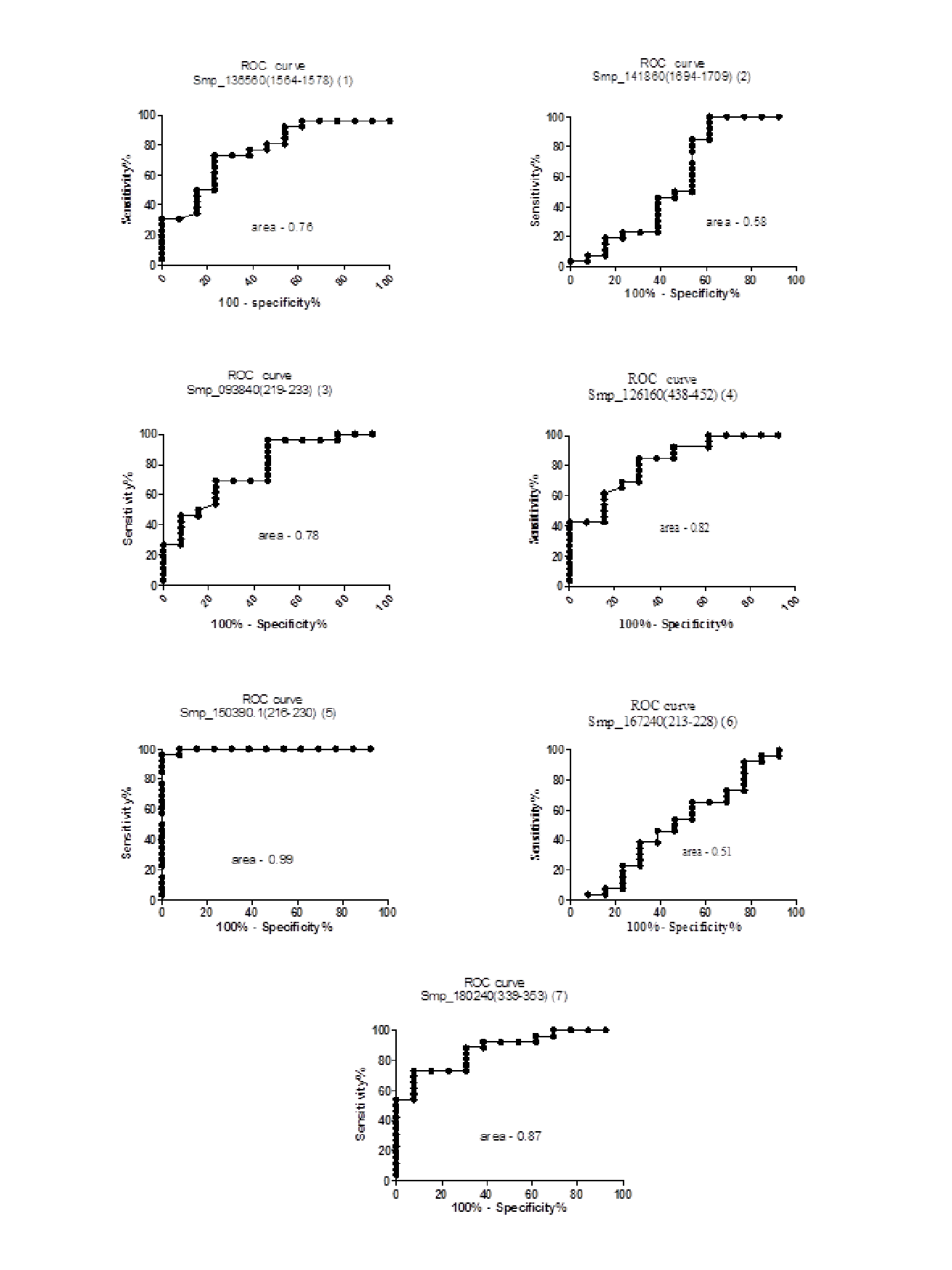

Supplement: S2 Fig — (TIF) [file pone.0182299.s002.tif]

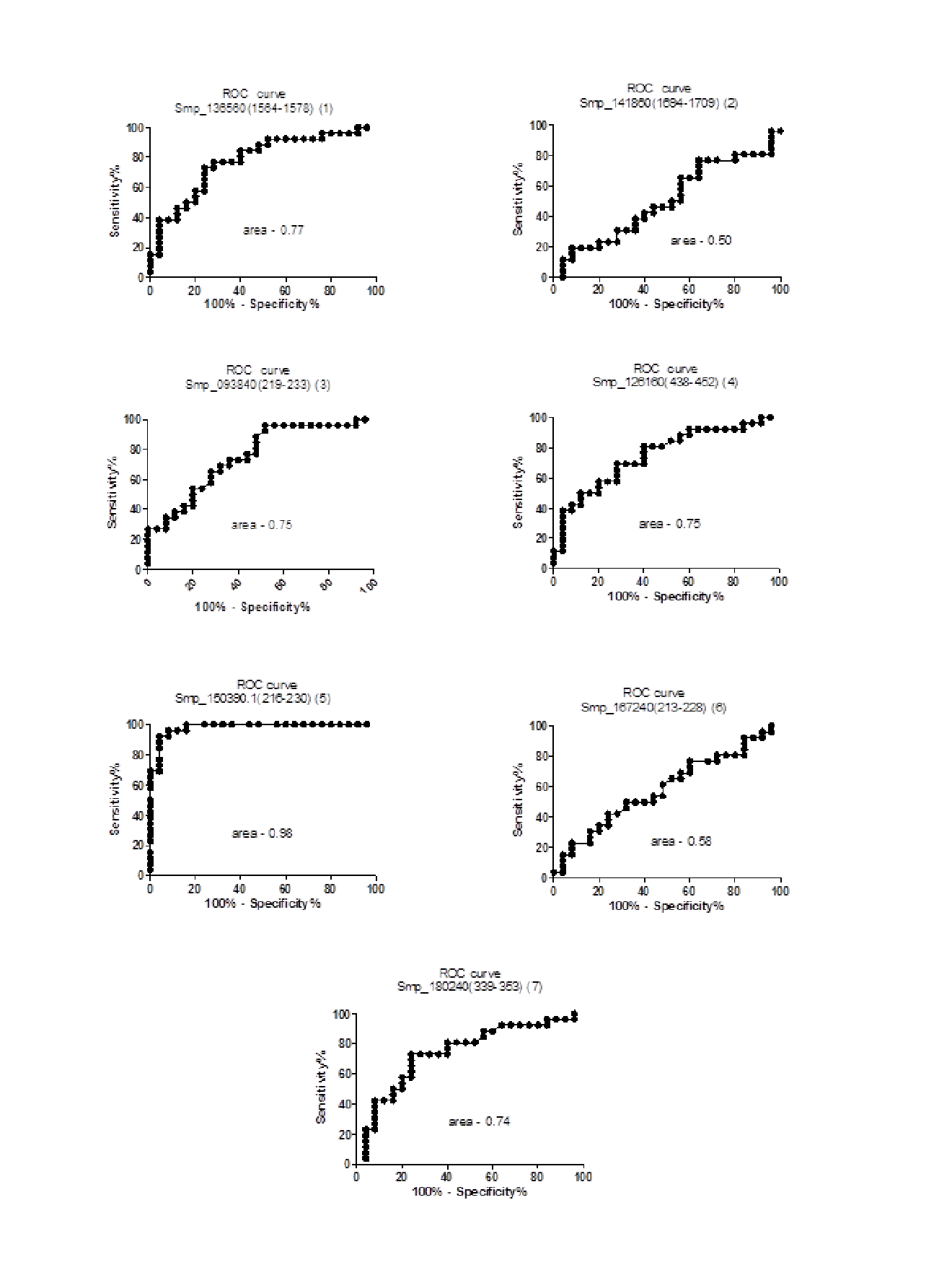

Supplement: S3 Fig — (TIF) [file pone.0182299.s003.tif]
