# Supplementary material for: Selecting targets for the diagnosis of Schistosoma mansoni infection: An integrative approach using multi-omic and immunoinformatics data
Source: PLoS One. 2017 Aug 17;12(8):e0182299. doi: 10.1371/journal.pone.0182299 (PMC5560627; doi:10.1371/journal.pone.0182299)
Supplement: S1 Table — aLife stages of parasite in definitive host. 1—schistosomulum, lung chistosomulum, adult worm and egg. 2—schistosomulum, lung schistosomulum and adult worm. (DOCX) [file pone.0182299.s004.docx]

**Table S1. *Schistosoma mansoni* proteins selected after *in silico* analysis according to the strategy outlined in the workflow.**

| **ID** | **Function predicted** | **Amino acids**  **lenght** | **Life stages ^a^** | **Number of alleles** |
| --- | --- | --- | --- | --- |
| Smp_136560 | expressed protein | 1995 | 1 | 16 |
| Smp_141860 | heat containing protein, putative | 4619 | 1 | 16 |
| Smp_017730 | 200-kDa GPI-anchored surface glycoprotein | 1656 | 1 | 15 |
| Smp_035190.2 | ras GTP exchange factor, son of sevenless, putative | 1568 | 1 | 15 |
| Smp_085540.6 | myosin heavy chain, putative | 1937 | 1 | 15 |
| Smp_130040 | pecanex-related protein | 2223 | 1 | 15 |
| Smp_131910 | surface protein, putative | 1680 | 1 | 15 |
| Smp_150390.2 | expressed protein | 847 | 1 | 15 |
| Smp_165800 | ATP-binding cassette transporter, putative | 2009 | 1 | 15 |
| Smp_174960 | merlin/moesin/ezrin/radixin, putative | 1028 | 1 | 15 |
| Smp_005860.3 | expressed protein | 408 | 1 | 14 |
| Smp_005860.4 | expressed protein | 408 | 1 | 14 |
| Smp_045360.3 | expressed protein | 1226 | 1 | 14 |
| Smp_050110 | gamma-secretase subunit aph-1, putative | 320 | 1 | 14 |
| Smp_076950.2 | solute carrier family 33 (acetyl-CoA transporter) putative | 696 | 1 | 14 |
| Smp_097090.2 | expressed protein | 686 | 1 | 14 |
| Smp_097090.3 | expressed protein | 686 | 1 | 14 |
| Smp_125550 | expressed protein | 1412 | 1 | 14 |
| Smp_137070.1 | expressed protein | 550 | 1 | 14 |
| Smp_137070.2 | expressed protein | 659 | 1 | 14 |
| Smp_142550 | nuclear pore membrane glycoprotein gp210- related | 2192 | 1 | 14 |
| Smp_145900 | adam, putative | 862 | 1 | 14 |
| Smp_146590.2 | expressed protein | 1225 | 1 | 14 |
| Smp_175360.2 | cation-transporting atpase worm, putative | 1509 | 1 | 14 |
| Smp_033760.1 | expressed protein | 570 | 1 | 13 |
| Smp_034940.1 | Protein C10orf118 (CTCL tumor antigen HD-CL- 01/L14-2), putative | 776 | 1 | 13 |
| Smp_034940.2 | Protein C10orf118 (CTCL tumor antigen HD-CL- 01/L14-2), putative | 784 | 1 | 13 |
| Smp_099150 | expressed protein | 239 | 1 | 13 |
| Smp_140450.1 | cleavage and polyadenylation specificity factor, putative | 1061 | 1 | 13 |
| Smp_141090.1 | Protein C10orf118 (CTCL tumor antigen HD-CL- 01/L14-2), putative | 686 | 1 | 13 |
| Smp_153310.2 | lethal giant larva homologue, putative | 919 | 1 | 13 |
| Smp_155570 | hypothetical protein | 474 | 1 | 13 |
| Smp_173060 | pak-interacting exchange factor, beta-pix/cool- 1, putative | 749 | 1 | 13 |
| Smp_179370 | low-density lipoprotein receptor (ldl), putative | 836 | 1 | 13 |
| Smp_070240 | venom allergen-like (VAL) 7 protein | 193 | 2 | 13 |
| Smp_093840 | trispanning orphan receptor; TORE, putative | 239 | 2 | 14 |
| Smp_126160 | poly(p)/ATP NAD kinase, putative | 1077 | 2 | 14 |
| Smp_150390.1 | expressed protein | 668 | 2 | 14 |
| Smp_167240 | expressed protein | 776 | 2 | 14 |
| Smp_180240 | f-spondin, putative | 941 | 2 | 14 |

^a^Life stages of parasite in definitive host

1 - schistosomulum, lung chistosomulum, adult worm and egg

2 - schistosomulum, lung schistosomulum and adult worm
