# Supplementary material for: Selecting targets for the diagnosis of Schistosoma mansoni infection: An integrative approach using multi-omic and immunoinformatics data
Source: PLoS One. 2017 Aug 17;12(8):e0182299. doi: 10.1371/journal.pone.0182299 (PMC5560627; doi:10.1371/journal.pone.0182299)
Supplement: S2 Table — aCI—confidence interval; bPPV = positive predictive value; PPV = number of true positives/(number of true positives + number of false positives); cNPV—negative predictive value; NPV = number of true negatives/(number of true negatives + number of false negatives). (DOCX) [file pone.0182299.s005.docx]

**S2 Table. Performance of ELISA using selected peptides and sera from healthy donors as negative control.**

| Peptide number | Synthetic peptide | Area under ROC curve (SD) | CI^a^ | P value | Cutoff  value | Sensitivity | Specificity | PPV^b^ | NPV^c^ |
| --- | --- | --- | --- | --- | --- | --- | --- | --- | --- |
| 1 | Smp_136560 (1564-1578) | 0.76 (0.081) | 0.6024-0.9213 | 0.0084 | 0.1098 | 73.08% | 76.92% | 86,36% | 58,82% |
| 2 | Smp_141860 (1694-1709) | 0.58 (0.11) | 0.316-0.8041 | 0.4042 | 0.0562 | 84.62% | 46.15% | 75% | 60% |
| 3 | Smp_093840(219-233) | 0.78 (0.078) | 0.6277-0.9374 | 0.0044 | 0.295 | 69.23% | 76.92% | 85,71% | 55,5% |
| 4 | Smp_126160(438-452) | 0.82 (0.069) | 0.6871-0.9609 | 0.0011 | 0,2118 | 84.62% | 69.23% | 84,72% | 69,23% |
| 5 | Smp_150390.1(216-230) | 0.99 (0.004) | 0.9874-1.007 | <0.0001 | 0.244 | 96.15% | 100% | 96,15% | 100% |
| 6 | Smp_167240(213 -228) | 0.51(0.105) | 0.3068-0.7198 | 0.8934 | 0.1848 | 53.85% | 53.85% | 63,15% | 30% |
| 7 | Smp_180240(339-353) | 0.87 (0.055) | 0.7633-0.9823 | 0.0002 | 0.556 | 73.08% | 92.31% | 95% | 63,15% |

^a^CI- confidence interval

^b^PPV - positive predictive value - PPV= Number of true positives / (number of true positives + number of false positives)

^c^NPV - negative predictive value - NPV= Number of true negatives / (number of true negatives + number of false negatives)
