# Supplementary material for: Selecting targets for the diagnosis of Schistosoma mansoni infection: An integrative approach using multi-omic and immunoinformatics data
Source: PLoS One. 2017 Aug 17;12(8):e0182299. doi: 10.1371/journal.pone.0182299 (PMC5560627; doi:10.1371/journal.pone.0182299)
Supplement: S3 Table — aCI—confidence interval; bPPV = positive predictive value; PPV = number of true positives/(number of true positives + number of false positives); cNPV—negative predictive value; NPV = number of true negatives/(number of true negatives + number of false negatives). (DOCX) [file pone.0182299.s006.docx]

**S3 Table. Performance of ELISA using selected peptides and sera from non-infected individuals living in endemic area as negative control.**

| Peptide number | Synthetic peptide | Area under ROC curve (SD) | CI^a^ | P value | Cutoff  value | Sensitivity | Specificity | PPV^b^ | NPV^c^ |
| --- | --- | --- | --- | --- | --- | --- | --- | --- | --- |
| 1 | Smp_136560 (1564-1578) | 0.77 (0.065) | 0.6502-0.9067 | 0.0006 | 0.1033 | 76.92% | 72% | 74% | 75% |
| 2 | Smp_141860 (1694-1709) | 0.50 (0.082) | 0.3419-0.6643 | 0.969 | 0.098 | 53% | 44% | 50% | 47% |
| 3 | Smp_093840(219-233) | 0.75 (0.067) | 0.623-0.8878 | 0.001 | 0.342 | 46.15% | 80% | 70% | 58% |
| 4 | Smp_126160(438-452) | 0.75 (0.068) | 0.6175-0.8856 | 0.002 | 0.262 | 69.23% | 72% | 72% | 69% |
| 5 | Smp_150390.1(216-230) | 0.98 (0.014) | 0.9524-1.011 | <0.0001 | 0.262 | 92.31% | 96% | 96% | 92% |
| 6 | Smp_167240(213 -228) | 0.58 (0.080) | 0.432-0.7464 | 0.274 | 0.155 | 53.85% | 56% | 56% | 53% |
| 7 | Smp_180240(339-353) | 0.74 (0.070) | 0.6072-0.8836 | 0.002 | 0.554 | 73.8% | 76% | 76% | 73% |

^a^CI- confidence interval

^b^PPV - positive predictive value - PPV= Number of true positives / (number of true positives + number of false positives)

^c^NPV - negative predictive value - NPV= Number of true negatives / (number of true negatives + number of false negatives)
