# Supplementary material for: Selecting targets for the diagnosis of Schistosoma mansoni infection: An integrative approach using multi-omic and immunoinformatics data
Source: PLoS One. 2017 Aug 17;12(8):e0182299. doi: 10.1371/journal.pone.0182299 (PMC5560627; doi:10.1371/journal.pone.0182299)
Supplement: S4 Table — Nd—not determined. O.D.—optical density at 560nm. *—serum from patient evalueted before treatament in the infected group (INF). nd—not determined. r—antigen-reactive sera. (DOCX) [file pone.0182299.s007.docx]

**S4 Table. Description of parasitological, molecular and serological results from each serum sample analyzed in the study**.

| Serum  code | Parasitological and molecular methodology | | | | | | Peptides absorbance level in ELISA assay | | | | | | |
| --- | --- | --- | --- | --- | --- | --- | --- | --- | --- | --- | --- | --- | --- |
|  | Kato -Katz | | | | TF-  Test® | PCR-ELISA | 1  (O.D) | 2  (O.D) | 3  (O.D) | 4  (O.D) | 5  (O.D) | 6  (O.D) | 7  (O.D) |
|  | 1ºsample  (12 slides)  opg | 2º sample  (2 slides)  opg | 3ºsample  (2 slides)  opg | 4ºsample  (2 slides)  opg |  |  |  |  |  |  |  |  |  |
| INF-1 | 0 | 0 | 0 | 0 | pos | pos | 0.3175^r^ | 0.2695^r^ | 0.4510^r^ | 0.5670^r^ | 0.3530^r^ | 0.1985^r^ | 0.8010^r^ |
| INF-2 | 0 | 0 | 12 | 0 | 0 | neg | 0.1430^r^ | 0.0950^r^ | 0.3400^r^ | 0.3985^r^ | 0.3510^r^ | 0.1520 | 1.5420^r^ |
| INF-3 | 0 | 0 | 12 | 0 | 0 | neg | 0.1755^r^ | 0.0520 | 0.2795 | 0.2040 | 0.3315^r^ | 0.0440 | 0.4640 |
| INF- 4 | 0 | 0 | 0 | 0 | pos | neg | 0.0675 | 0.0385 | 0.2695 | 0.1775 | 0.2995^r^ | 0.0775 | 0.9170^r^ |
| INF-5 | 0 | 0 | 0 | 0 | pos | neg | 0.1530^r^ | 0.0870^r^ | 0.3630^r^ | 0.2740^r^ | 0.3245^r^ | 0.0825 | 0.8800^r^ |
| INF-6 | 0 | 0 | 0 | 0 | pos | neg | 0.3755^r^ | 0.2660^r^ | 0.5770^r^ | 0.6350^r^ | 0.3145^r^ | 0.4580^r^ | 0.4600 |
| INF-7 | 0 | 0 | 0 | 12 | 0 | pos | 0.0815 | 0.0250 | 0.2605 | 0.1630 | 0.2660^r^ | 0.0920 | 0.4320 |
| INF-8 | 0 | 0 | 0 | 12 | 0 | pos | 0.1215^r^ | 0.0735^r^ | 0.2735 | 0.3000^r^ | 0.2865^r^ | 0.1460 | 0.8210^r^ |
| INF-9 | 1 | 0 | 0 | 0 | 0 | pos | 0.0900 | 0.0645^r^ | 0.3060^r^ | 0.2365^r^ | 0.3060^r^ | 0.1305 | 1.0580^r^ |
| INF-10 | 0 | 0 | 0 | 0 | pos | neg | 0.2100^r^ | 0.1325^r^ | 0.4685^r^ | 0.3955^r^ | 0.3145^r^ | 0.1845 | 0.6540^r^ |
| INF-11 | 0 | 0 | 0 | 0 | pos | neg | 0.4615^r^ | 0.2880^r^ | 0.5585^r^ | 0.7375^r^ | 0.3490^r^ | 0.3710^r^ | 0.7730^r^ |
| INF-12 | 0 | 0 | 60 | 0 | 0 | neg | 0.1115^r^ | 0.0585^r^ | 0.3725^r^ | 0.2360^r^ | 0.2665^r^ | 0.1180 | 0.7740^r^ |
| INF-13 | 0 | 0 | 0 | 0 | pos | neg | 0.1835^r^ | 0.1210^r^ | 0.3660^r^ | 0.3460^r^ | 0.3445^r^ | 0.1520 | 0.8580^r^ |
| INF-14 | 1 | 0 | 0 | 0 | 0 | pos | 0.3340^r^ | 0.1960^r^ | 0.3765^r^ | 0.5390^r^ | 0.3260^r^ | 0.3790^r^ | 1.1980^r^ |
| INF-15 | 10 | 0 | 0 | 12 | pos | pos | 0.1040 | 0.0380 | 0.2655 | 0.2130^r^ | 0.2705^r^ | 0.2330^r^ | 0.6040^r^ |
| INF-16 | 14 | 0 | 0 | 12 | pos | pos | 0.1305^r^ | 0.0785^r^ | 0.3080^r^ | 0.2940^r^ | 0.2750^r^ | 0.1960^r^ | 1.3540^r^ |
| INF-17 | 0 | 12 | 0 | 0 | 0 | pos | 0.2750^r^ | 0.1410^r^ | 0.3085^r^ | 0.4300^r^ | 0.3555^r^ | 0.2675^r^ | 1.0220^r^ |
| INF-18 | 0 | 0 | 0 | 0 | pos | neg | 0.1460^r^ | 0.0735^r^ | 0.2960 | 0.3550^r^ | 0.3270^r^ | 0.2590^r^ | 0.9880^r^ |
| INF-19 | 0 | 0 | 0 | 0 | pos | pos | 0.2380^r^ | 0.1465^r^ | 0.3395^r^ | 0.5305^r^ | 0.3540^r^ | 0.3165^r^ | 0.6770^r^ |
| INF-20 | 0 | 0 | 60 | 0 | 0 | neg | 0.2725^r^ | 0.1625^r^ | 0.4755^r^ | 0.4135^r^ | 0.3505^r^ | 0.3560^r^ | 0.5680^r^ |
| INF-21 | 0 | 0 | 24 | 0 | 0 | neg | 0.2770^r^ | 0.1415^r^ | 0.4055^r^ | 0.4600^r^ | 0.3505^r^ | 0.3045^r^ | 0.7220^r^ |
| INF-22 | 0 | 0 | 0 | 0 | 0 | pos | 0.1165^r^ | 0.1715^r^ | 0.2590 | 0.2775^r^ | 0.3400^r^ | 0.1560 | 0.5155 |
| INF-23 | 0 | 0 | 0 | 0 | 0 | pos | 0.0705 | 0.1735^r^ | 0.2675 | 0.1350 | 0.2655^r^ | 0.0785 | 0.3090 |
| INF-24 | 0 | 0 | 0 | 0 | 0 | pos | 0.0455 | 0.0905^r^ | 0.1960 | 0.3880^r^ | 0.2665^r^ | 0.0515 | 0.2160 |
| INF-25 | 0 | 0 | 0 | 0 | 0 | pos | 0.2800^r^ | 0.4900^r^ | 0.5850^r^ | 0.2725^r^ | 0.2550^r^ | 0.1170 | 0.6980^r^ |
| INF-26 | 0 | 0 | 0 | 0 | 0 | pos | 0.0090 | 0.2615^r^ | 0.3440^r^ | 0.2410^r^ | 0.2195 | 0.1905^r^ | 0.5080 |
| NEG-1 | 0 | 0 | 0 | 0 | 0 | neg | 0.1995^r^ | 0.1940^r^ | 0.3460^r^ | 0.0920 | 0.2240 | 0.2470^r^ | 0.4460 |
| NEG-2 | 0 | 0 | 0 | 0 | 0 | neg | 0.0640 | 0.1025^r^ | 0.2335 | 0.2010 | 0.1590 | 0.0875 | 0.3645 |
| NEG-3 | 0 | 0 | 0 | 0 | 0 | neg | 0.0460 | 0.0715^r^ | 0.1870 | 0.1680 | 0.1440 | 0.1630 | 0.5185 |
| NEG-4 | 0 | 0 | 0 | 0 | 0 | neg | 0.1330^r^ | 0.2355^r^ | 0.3180^r^ | 0.2230^r^ | 0.2190 | 0.1660 | 0.5175 |
| NEG-5 | 0 | 0 | 0 | 0 | 0 | neg | 0.0770 | 0.1165^r^ | 0.2995^r^ | 0.2805^r^ | 0.1980 | 0.0475 | 0.4660 |
| NEG-6 | 0 | 0 | 0 | 0 | 0 | neg | 0.0385 | 0.0600^r^ | 0.2250 | 0.1760 | 0.1710 | 0.1655 | 0.4890 |
| NEG-7 | 0 | 0 | 0 | 0 | 0 | neg | 0.0975 | 0.1940^r^ | 0.3645^r^ | 0.2105 | 0.2170 | 0.1260 | 0.5245 |
| NEG-8 | 0 | 0 | 0 | 0 | 0 | neg | 0.1000 | 0.1600^r^ | 0.2855 | 0.1770 | 0.2090 | 0.2765^r^ | 0.9420^r^ |
| NEG-9 | 0 | 0 | 0 | 0 | 0 | neg | 0.0690 | 0.0745^r^ | 0.3855^r^ | 0.3175^r^ | 0.2190 | 0.1110 | 0.7270^r^ |
| NEG-10 | 0 | 0 | 0 | 0 | 0 | neg | 0.1095 | 0.1925^r^ | 0.2015 | 0.2305^r^ | 0.2260 | 0.1460 | 0.4320 |
| NEG-11 | 0 | 0 | 0 | 0 | 0 | neg | 0.0535 | 0.0680^r^ | 0.2170 | 0.1035 | 0.1850 | 0.1035 | 0.4880 |
| NEG-12 | 0 | 0 | 0 | 0 | 0 | neg | 0.0450 | 0.0615^r^ | 0.1975 | 0.2415^r^ | 0.1640 | 0.2765^r^ | 0.0580 |
| NEG-13 | 0 | 0 | 0 | 0 | 0 | neg | 0.1740^r^ | 0.1495^r^ | 0.2510 | 0.3625^r^ | 0.1960 | 0.0860 | 0.7890^r^ |
| NEG-14 | 0 | 0 | 0 | 0 | 0 | neg | 0.1025 | 0.1895^r^ | 0.2605 | 0.1765 | 0.1730 | 0.2300^r^ | 0.6795^r^ |
| NEG-15 | 0 | 0 | 0 | 0 | 0 | neg | 0.0360 | 0.0390 | 0.2535 | 0.1525 | 0.1520 | 0.1310 | 0.2700 |
| NEG-16 | 0 | 0 | 0 | 0 | 0 | neg | 0.0670 | 0.1260^r^ | 0.2715 | 0.2515^r^ | 0.1970 | 0.3295^r^ | 0.2340 |
| NEG-17 | 0 | 0 | 0 | 0 | 0 | neg | 0.0540 | 0.0590^r^ | 0.1805 | 0.1740 | 0.1450 | 0.1905^r^ | 0.3260 |
| NEG-18 | 0 | 0 | 0 | 0 | 0 | neg | 0.1950^r^ | 0.4420^r^ | 0.3085^r^ | 0.3915^r^ | 0.1960 | 0.3940^r^ | 0.2620 |
| NEG-19 | 0 | 0 | 0 | 0 | 0 | neg | 0.1470^r^ | 0.2095^r^ | 0.3685^r^ | 0.3180^r^ | 0.1930 | 0.0410 | 0.3795 |
| NEG-20 | 0 | 0 | 0 | 0 | 0 | neg | 0.0035 | 0.0885^r^ | 0.2745 | 0.1705 | 0.1700 | 0.1110 | 0.3665 |
| NEG-21 | 0 | 0 | 0 | 0 | 0 | neg | 0.0000 | 0.0925^r^ | 0.2495 | 0.1415 | 0.1980 | 0.0710 | 0.3455 |
| NEG-22 | 0 | 0 | 0 | 0 | 0 | neg | 0.0420 | 0.2270^r^ | 0.2765 | 0.2870^r^ | 0.2150 | 0.1540 | 0.5400 |
| NEG-23 | 0 | 0 | 0 | 0 | 0 | neg | 0.0765 | 0.0765^r^ | 0.2225 | 0.2480^r^ | 0.1720 | 0.0835 | 0.8005^r^ |
| NEG-24 | 0 | 0 | 0 | 0 | 0 | neg | 0.2860^r^ | 0.2335^r^ | 0.3875^r^ | 0.5450^r^ | 0.2820^r^ | 0.1925^r^ | 1.5420^r^ |
| NEG-25 | 0 | 0 | 0 | 0 | 0 | neg | 0.0495 | 0.0210 | 0.2560 | 0.2130^r^ | 0.2600^r^ | 0.0730 | 0.4640 |
| 30DTP-1 | 0 | 0 | 0 | 0 | 0 | nd | 0.0710 | 0.0405 | 0.2060 | 0.2305^r^ | 0.1260 | 0.1710 | 0.7880^r^ |
| 30DTP-2* | 0 | 0 | 0 | 0 | 0 | nd | 0.1360^r^ | 0.0700^r^ | 0.3200^r^ | 0.4290^r^ | 0.2390 | 0.3305^r^ | 0.5920^r^ |
| 30DTP-3* | 0 | 0 | 0 | 0 | 0 | nd | 0.0470 | 0.0195 | 0.2540 | 0.1780 | 0.1970 | 0.1120 | 0.9900^r^ |
| 30DTP-4* | 0 | 0 | 0 | 0 | 0 | nd | 0.0380 | 0.0155 | 0.1090 | 0.1510 | 0.1220 | 0.1240 | 0.7690^r^ |
| 30DTP-5* | 0 | 0 | 0 | 0 | 0 | nd | 0.1415^r^ | 0.0570^r^ | 0.3085^r^ | 0.3050^r^ | 0.2320 | 0.2230^r^ | 0.5520 |
| 30DTP-6 | 0 | 0 | 0 | 0 | 0 | nd | 0.0925 | 0.0400 | 0.2335 | 0.2890^r^ | 0.2000 | 0.1875^r^ | 0.3950 |
| 30DTP-7 | 0 | 0 | 0 | 0 | 0 | nd | 0.0560 | 0.0250 | 0.3505^r^ | 0.2315^r^ | 0.2290 | 0.2050^r^ | 0.3060 |
| 30DTP-8* | 0 | 0 | 0 | 0 | 0 | nd | 0.0700 | 0.0315 | 0.2590 | 0.1855 | 0.1760 | 0.1360 | 0.7590^r^ |
| 30DTP-9 | 0 | 0 | 0 | 0 | 0 | nd | 0.0980 | 0.0520 | 0.2980^r^ | 0.2590^r^ | 0.1600 | 0.2390^r^ | 0.5270 |
| 30DTP-10* | 0 | 0 | 0 | 0 | 0 | nd | 0.0500 | 0.0270 | 0.2550 | 0.1645 | 0.2030 | 0.1405 | 0.4520 |
| 30DTP-11 | 0 | 0 | 0 | 0 | 0 | nd | 0.0800 | 0.0378 | 0.2593 | 0.2423^r^ | 0.1885 | 0.1868^r^ | 0.6130^r^ |
| 30DTP-12* | 0 | 0 | 0 | 0 | 0 | nd | 0.1085 | 0.0610^r^ | 0.2605 | 0.1995 | 0.1330 | 0.1445 | 0.7810^r^ |
| 30DTP-13* | 0 | 0 | 0 | 0 | 0 | nd | 0.1115^r^ | 0.0700^r^ | 0.2920 | 0.2760^r^ | 0.2260 | 0.2105^r^ | 0.5640^r^ |
| 30DTP-14 | 0 | 0 | 0 | 0 | 0 | nd | 0.2685^r^ | 0.1960^r^ | 0.3570^r^ | 0.7425^r^ | 0.1700 | 0.3815^r^ | 0.4350 |
| 30DTP-15* | 0 | 0 | 0 | 0 | 0 | nd | 0.2550^r^ | 0.1840^r^ | 0.3915^r^ | 0.3415^r^ | 0.2150 | 0.2670^r^ | 0.4190 |
| 30DTP-16* | 0 | 0 | 0 | 0 | 0 | nd | 0.1180^r^ | 0.0500 | 0.3410^r^ | 0.3045^r^ | 0.1730 | 0.2240^r^ | 0.4860 |
| 30DTP-17 | 0 | 0 | 0 | 0 | 0 | nd | 0.2825^r^ | 0.1250^r^ | 0.3610^r^ | 0.3775^r^ | 0.1950 | 0.4200^r^ | 0.5000 |
| 180DTP-1 | 0 | 0 | 0 | 0 | 0 | nd | 0.0605 | 0.0390 | 0.256 | 0.1915 | 0.1130 | 0.1775 | 0.5455 |
| 180DTP-2 | 0 | 0 | 0 | 0 | 0 | nd | 0.2190^r^ | 0.1380^r^ | 0.380^r^ | 0.3895^r^ | 0.2645^r^ | 0.4210^r^ | 0.8060^r^ |
| 180DTP-3 | 0 | 0 | 0 | 0 | 0 | nd | 0.0545 | 0.0395 | 0.308^r^ | 0.1815 | 0.2090 | 0.2450^r^ | 0.6500^r^ |
| 180DTP-4* | 0 | 0 | 0 | 0 | 0 | nd | 0.0385 | 0.1220^r^ | 0.183 | 0.1805 | 0.1108 | 0.0725 | 0.4980 |
| 180DTP-5* | 0 | 0 | 0 | 0 | 0 | nd | 0.0700 | 0.0745^r^ | 0.236 | 0.2260^r^ | 0.1348 | 0.1175 | 0.9250^r^ |
| 180DTP-6* | 0 | 0 | 0 | 0 | 0 | nd | 0.0950 | 0.1030^r^ | 0.276 | 0.2880^r^ | 0.1513 | 0.1745 | 0.7200^r^ |
| 180DTP-7* | 0 | 0 | 0 | 0 | 0 | nd | 0.1515^r^ | 0.1985^r^ | 0.410^r^ | 0.4715^r^ | 0.1018 | 0.3050^r^ | 0.4080 |
| 180DTP-8* | 0 | 0 | 0 | 0 | 0 | nd | 0.1770^r^ | 0.2050^r^ | 0.333^r^ | 0.4110^r^ | 0.1268 | 0.2060^r^ | 0.5020 |
| 180DTP-9* | 0 | 0 | 0 | 0 | 0 | nd | 0.1795^r^ | 0.1705^r^ | 0.383^r^ | 0.5070^r^ | 0.1078 | 0.2775^r^ | 0.8205^r^ |

nd - not determined

O.D. - optical density at 560nm

* - serum from patient evaluated before treatment in the infected group (INF

^r^  - antigen-reactive sera
